# Multiple functions of G protein-coupled receptor kinases

**DOI:** 10.1186/1750-2187-9-1

**Published:** 2014-03-06

**Authors:** Kenji Watari, Michio Nakaya, Hitoshi Kurose

**Affiliations:** 1 Department of Pharmacology and Toxicology, Graduate School of Pharmaceutical Sciences, Kyushu University, 3-1-1 Maidashi, Higashi-ku, Fukuoka 812-8582, Japan

**Keywords:** G protein-coupled receptor (GPCR), G protein-coupled receptor kinase (GRK), Cell signaling, Biased agonist

## Abstract

Desensitization is a physiological feedback mechanism that blocks detrimental effects of persistent stimulation. G protein-coupled receptor kinase 2 (GRK2) was originally identified as the kinase that mediates G protein-coupled receptor (GPCR) desensitization. Subsequent studies revealed that GRK is a family composed of seven isoforms (GRK1–GRK7). Each GRK shows a differential expression pattern. GRK1, GRK4, and GRK7 are expressed in limited tissues. In contrast, GRK2, GRK3, GRK5, and GRK6 are ubiquitously expressed throughout the body. The roles of GRKs in GPCR desensitization are well established. When GPCRs are activated by their agonists, GRKs phosphorylate serine/threonine residues in the intracellular loops and the carboxyl-termini of GPCRs. Phosphorylation promotes translocation of β-arrestins to the receptors and inhibits further G protein activation by interrupting receptor-G protein coupling. The binding of β-arrestins to the receptors also helps to promote receptor internalization by clathrin-coated pits. Thus, the GRK-catalyzed phosphorylation and subsequent binding of β-arrestin to GPCRs are believed to be the common mechanism of GPCR desensitization and internalization. Recent studies have revealed that GRKs are also involved in the β-arrestin-mediated signaling pathway. The GRK-mediated phosphorylation of the receptors plays opposite roles in conventional G protein- and β-arrestin-mediated signaling. The GRK-catalyzed phosphorylation of the receptors results in decreased G protein-mediated signaling, but it is necessary for β-arrestin-mediated signaling. Agonists that selectively activate GRK/β-arrestin-dependent signaling without affecting G protein signaling are known as β-arrestin-biased agonists. Biased agonists are expected to have potential therapeutic benefits for various diseases due to their selective activation of favorable physiological responses or avoidance of the side effects of drugs. Furthermore, GRKs are recognized as signaling mediators that are independent of either G protein- or β-arrestin-mediated pathways. GRKs can phosphorylate non-GPCR substrates, and this is found to be involved in various physiological responses, such as cell motility, development, and inflammation. In addition to these effects, our group revealed that GRK6 expressed in macrophages mediates the removal of apoptotic cells (engulfment) in a kinase activity-dependent manner. These studies revealed that GRKs block excess stimulus and also induce cellular responses. Here, we summarized the involvement of GRKs in β-arrestin-mediated and G protein-independent signaling pathways.

## Introduction

G protein-coupled receptor kinases (GRKs) were originally identified as the kinases that phosphorylate and desensitize agonist-bound G protein-coupled receptors (GPCRs) [1]. The phosphorylation of agonist-bound GPCR by GRKs leads to the translocation and binding of β-arrestins to the receptors, inhibiting further G protein activation by blocking receptor-G protein coupling [2,3]. The phosphorylation of GPCR by GRKs and the binding of β-arrestins to the receptors also promote agonist-bound GPCR internalization [4-6]. Thus, the GRK-catalyzed phosphorylation and binding of β-arrestin to the receptors are believed to be the common mechanism of GPCR desensitization [7,8]. GPCR desensitization is important for maintaining homeostasis, as malfunction of the desensitization process could cause various diseases such as heart failure [9-11], inappropriate diuresis [12], asthma [13], Parkinson’s disease [14], and autoimmune disease [15]. Thus, GRKs play an essential role in maintaining cells and tissues in normal states.

GRKs are composed of seven isoforms (GRK1–GRK7) [16]. Although each GRK is involved in GPCR desensitization, some differences are observed in the expression, structure, and functions of GRKs [17,18]. GRK1, GRK4, and GRK7 are expressed in limited tissues. GRK1 and GRK7 are expressed in the retina [19-21], and GRK4 is expressed in the testis [22]. In contrast, other GRKs (GRK2, GRK3, GRK5, and GRK6) are expressed ubiquitously throughout the body [23-26]. Based on sequence homology, the GRK family can be divided into the three following subfamilies: the GRK1 subfamily composed of GRK1 and GRK7, the GRK2 subfamily composed of GRK2 and GRK3, and the GRK4 subfamily composed of GRK4, GRK5, and GRK6. All GRK isoforms share similar domains, which are composed of an amino-terminal domain unique to the GRK family of kinases, a regulator of G protein signaling homology domain; which could regulate GPCR signaling by phosphorylation-independent mechanisms [27-29], a serine/threonine protein kinase domain, and a carboxyl-terminal domain [30]. The amino-terminal domain of GRK2 interacts with the G protein βγ subunit, whereas that of GRK4, GRK5, and GRK6 interacts with phosphatidylinositol 4,5-bisphosphate (PIP2) [18,31,32]. Sequence divergence has been observed among GRKs in the carboxyl-terminal domain; GRK1 and GRK7 have short prenylation sequences [33], GRK2 and GRK3 have pleckstrin homology domains that interact with G protein βγ subunits [34,35] and PIP2 [36], and the members of the GRK4 subfamily have palmitoylation sites [22,37] and/or positively charged lipid-binding elements [38,39]. The carboxyl-termini of GRKs appear to be important for the localization and translocation of kinases to the membrane by means of posttranslational modifications or sites of interaction with lipids or membrane proteins [39]. The GRK4 subfamily (GRK4, GRK5, and GRK6) have been found to contain a functional nuclear localization signal (NLS) [39-41], and GRK5 and GRK6 have been shown to bind to DNA [40]. These properties could lead to functional diversification among GRKs. In fact, knockout mice for each GRK showed different phenotypes. GRK2 knockout mice are embryonic lethal [42], but knockout mice for other GRKs are born and develop normally. However, GRK6 knockout mice show dopaminergic supersensitivity [14] and develop autoimmune disease [43]. Further studies using knockout mice would reveal functional diversification among GRKs.

### Involvement of GRKs in G protein-independent signaling

**Figure 1:**
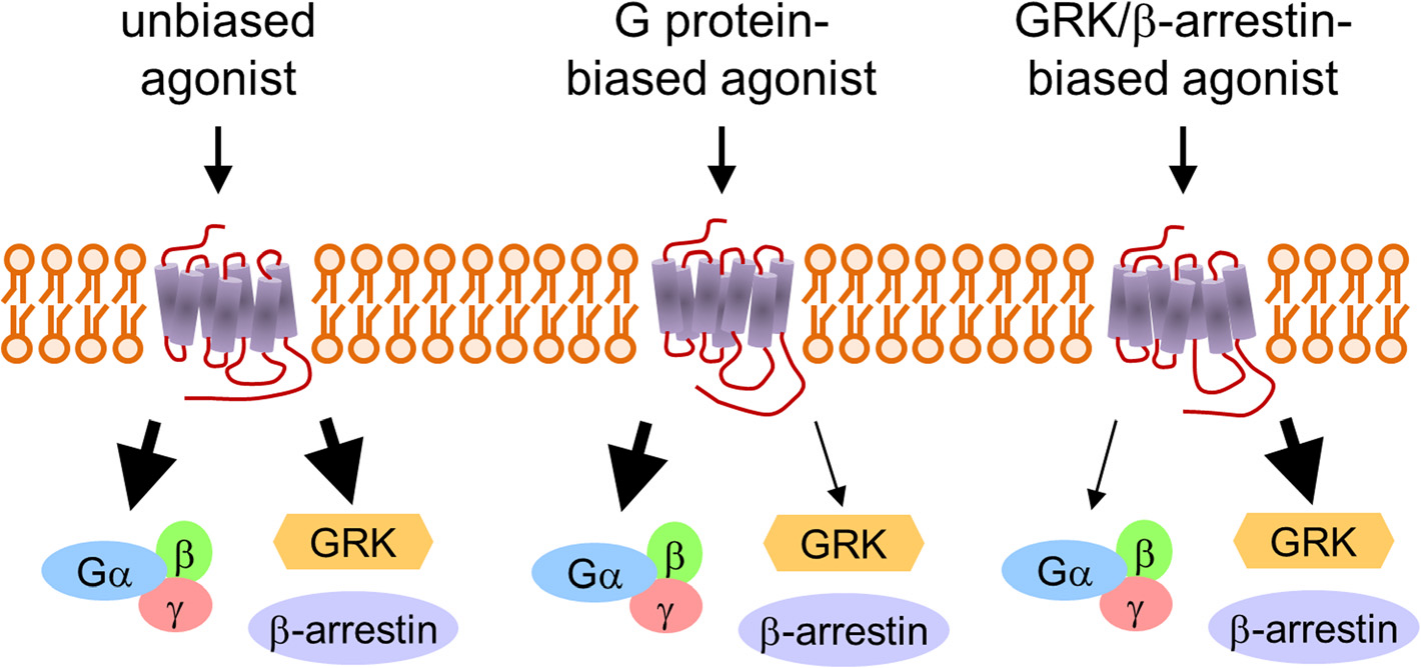
**GRKs are involved in cellular signaling that is independent of G protein activation.** Biased agonist activates either G protein signaling or GRK/β-arrestin-dependent signaling. Each agonist promotes distinct conformational changes of GPCRs. Unbiased agonists activate both G protein signaling and GRK/β-arrestin-dependent signaling, whereas biased agonists activate either G protein- or GRK/β-arrestin-dependent signaling as shown in bold arrows. Physiological responses mediated by GRK/β-arrestin-dependent signaling are believed to be distinct from those by G protein activation.

Recent studies have revealed that GRKs are involved not only in GPCR desensitization but also in G protein-independent signaling [44,45]. G protein-independent signaling requires GRKs and β-arrestins. GRK5 or GRK6 is required for G protein-independent extracellular signal-regulated kinase (ERK) activation by angiotensin II type 1A receptor (AT_1A_R) [46], vasopressin receptor 2 (V2R) [47], and β2-adrenergic receptor (β2-AR) [48]. GRK/β-arrestin-dependent signaling induces physiological responses that are different from G protein-mediated responses [49-51]. The activation of one of these signaling pathways could be beneficial, whereas the activation of the other signaling pathway could be harmful [52-55]. These findings have led to the identification and synthesis of agonists that selectively activate either G protein- or GRK/β-arrestin-dependent signaling [56,57]. Thus far, some agonists have been found to activate either G protein- [58] or GRK/β-arrestin-dependent signaling [59,60] by their own GPCRs. These agonists that can selectively activate only one signaling pathway are known as “biased agonists” [61] and have been proposed to be preferred for the treatment of various diseases [62]. As different conformational changes are induced in the cytoplasmic domain of GPCRs by the binding of full agonists and antagonists, biased agonists could induce the conformational state that selectively activates one of two signaling pathways [63] (Figure 1). However, the recent development of bioluminescent resonance energy transfer (BRET)-based G protein activation biosensors enabled the detection of G protein activation by stimulation with a GRK/β-arrestin-biased agonist [64]. It demonstrated that GRK/β-arrestin-biased agonists can activate G protein-mediated pathway, although the degree of activation is low. However, it is possible that the different conformational states of GPCRs selectively recruit a specific GRK, leading to the activation of GRK/β-arrestin-dependent signaling pathways.

The mechanism by which GRKs determine whether to promote GPCR desensitization or G protein-independent signaling remains unclear. Several studies have focused on the GRK subfamily that mediates desensitization or GRK/β-arrestin signaling [46,47,65,66]. It has been shown that the phosphorylation of AT_1A_R by GRK2 and GRK3 induces GPCR desensitization and internalization, whereas phosphorylation by GRK5 leads to β-arrestin-dependent ERK activation [46]. It has also been reported that GRK2 and GRK3 promote V2R desensitization, and GRK5 and GRK6 are responsible for the phosphorylation of ERK [47]. These studies demonstrate that different GRKs promote different functions of GPCRs, desensitization or signal transduction. Furthermore, the type of ligand is also important to determine whether to promote desensitization or signaling by GRKs. CC chemokine ligands 19 and 21 (CCL19 and CCL21) are the ligands of CC chemokine receptor type 7 (CCR7) that activate different GRK subfamilies, leading to receptor desensitization or signaling. CCL19 induces GPCR desensitization that was mediated by GRK3 and GRK6, whereas CCL21 promotes GRK/β-arrestin-mediated signaling that was dependent on GRK6 [65]. This result suggests that the ligands of GPCRs selectively activate specific GRKs, and activated GRKs then determine whether to promote GPCR desensitization or signaling. Although it is not fully understood how different ligands selectively recruit specific GRKs to the receptors, different conformational changes induced by different ligands may determine which GRK is selectively recruited to the receptors [63].

It has also been proposed that a differential phosphorylation pattern is essential for determining whether to promote GPCR desensitization or signaling. Butcher et al. found that different tissues and cells exhibit a differential GPCR phosphorylation pattern of the M3 muscarinic receptor [67]. However, they did not evaluate which kinases; such as protein kinase A, protein kinase C, and GRKs; are involved in the phosphorylation of the receptors. Nobles et al. demonstrated that different GRKs phosphorylate different sets of serine/threonine residues in the carboxyl-terminus of GPCR, and this determines whether desensitization or signaling is promoted by the receptor [68]. They found that GRK2 and GRK6 phosphorylate different sites in β2-AR, which determines the different functions of β-arrestin, β-arrestin-mediated desensitization or signaling [68]. Thus, the GPCR phosphorylation pattern (which is proposed as “phosphorylation barcoding”) [69] would be an important factor for the promotion of desensitization or signaling by GRKs.

Thus, the conformational changes of GPCRs and phosphorylation pattern of GPCRs could be important for G protein activation, GPCR desensitization, and GRK/β-arrestin-mediated signaling. Although “phosphorylation barcoding” was recently proposed as a key factor for determining whether to promote desensitization or GRK/β-arrestin-mediated signaling, it remains to be elucidated how each GRK phosphorylates specific serine/threonine residues. The identification of the consensus phosphorylation sequences for each GRK would be meaningful to understand how GRKs regulate GPCR desensitization and GRK/β-arrestin-dependent signaling.

### Physiological importance of GRK/β-arrestin-biased agonist

Many agonists can usually activate both G protein- and β-arrestin-mediated signaling pathways [62]. A biased agonist is defined as an agonist that selectively activates only one of these pathways [61]. Thus far, an increasing number of GPCR agonists have been found to function as biased agonists. It also suggests the potential use of biased agonists as a therapeutic agent [53,62]. Among various reports, biased agonists for β-ARs are well studied in terms of clinical use [70,71]. Noma et al. demonstrated that GRK/β-arrestin-biased signaling by β1-AR elicits cardioprotective effects in vivo [55]. GRK phosphorylates serine/threonine residues in the carboxyl-terminus of β1-AR. They substituted these serine/threonine residues with alanine and produced transgenic mice expressing mutant β1-AR in the heart (GRK^−^-β1-AR TG). They also produced transgenic mice expressing wild-type β1-AR in the heart (WT-β1-AR TG). When these mice were subjected to chronic exposure of isoproterenol, GRK^−^-β1-AR TG mice showed a significantly higher number of apoptotic cells than WT-β1-AR TG mice. This resulted in decreased cardiac performance in GRK^−^-β1-AR TG mice. They also demonstrated that epidermal growth factor receptor (EGFR) transactivation by GRK/β-arrestin-mediated, but not G protein-mediated, signaling is important for cardioprotective effects. As the chronic activation of Gs signaling by β1-AR is reported to be cardiotoxic, β-adrenergic blocking agents are beneficial for the treatment of heart failure [72]. They suggested that GRK/β-arrestin-biased agonists, which also antagonize Gs signaling, are more suitable for the treatment of heart failure. Among 20 β-adrenergic blocking agents, alprenolol and carvedilol have been identified as biased agonists for β1-AR [60], and carvedilol has been clinically used for the treatment of heart failure. Alprenolol and carvedilol could induce EGFR transactivation in a GRK/β-arrestin-dependent manner. However, it remains to be determined whether alprenolol-mediated G protein-independent signaling also has cardioprotective effects against heart failure. In contrast, our group recently reported that the long-term oral administration of metoprolol, a β-adrenergic blocking agent, induces cardiac fibrosis in mice by β1-AR in a GRK5/β-arrestin2-dependent manner without G protein activation [73]. Fibrosis is the excessive deposition of extracellular matrix, such as collagen and fibronectin, and is believed to be deleterious for cardiac function. In contrast to carvedilol and alprenolol, metoprolol does not promote the EGFR internalization and activation [60,73]. This suggests that metoprolol activates biased signaling in a different manner from that of carvedilol and alprenolol.

AT_1A_R has also been well studied as a model GPCR to analyze biased agonists [70,71]. Biased agonists that selectively activate GRK/β-arrestin-dependent signaling in cardiomyocytes have been reported to promote cardiomyocyte growth and cardiac hypertrophy and affect cardiac performance [74]. [Sar1, Ile4, Ile8] angiotensin II (SII), TRV120023, and TRV120027 have been developed as GRK/β-arrestin-biased agonists for AT_1A_R, and SII has been frequently used for the study of G protein-independent signaling of AT_1A_R [54,59,64,75,76]. Both SII and TRV120027 have been shown to increase cardiac contractility in vitro and in vivo [59,75]. In contrast, TRV120023 promotes the survival of cardiomyocytes during ischemia/reperfusion injury in vivo [54]. Thus, biased agonist-promoted GRK/β-arrestin-dependent signaling by AT_1A_R could be beneficial for the heart under physiological and pathological conditions. However, it remains to be determined which GRKs are involved in AT_1A_R-mediated biased signaling and which molecules downstream of GRKs and β-arrestins are responsible for signaling.

### Interaction of GRKs with non-GPCR proteins

**Figure 2:**
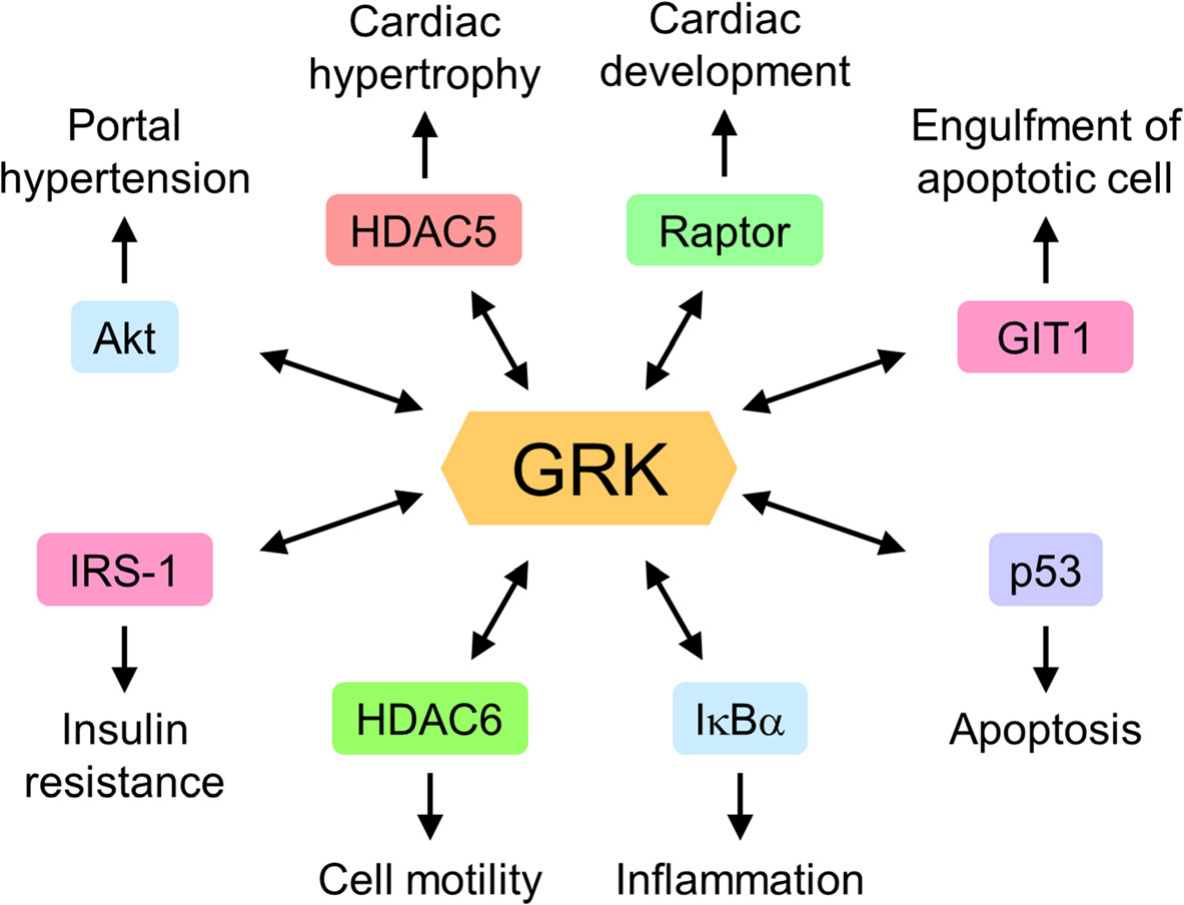
**Binding partners with GRKs.** GRKs regulate diverse signaling pathways by the interaction with intracellular proteins, resulting in various physiological responses.

In addition to the role of GRKs in GRK/β-arrestin-dependent signaling, it has been recognized that GRKs also interact with non-GPCR proteins [30,77]. Non-GPCR proteins that interact with GRKs include single-transmembrane receptors [78,79], cytosolic proteins [80-82], and nuclear proteins [83,84] (Figure 2). Many studies demonstrated that the interaction of GRKs with intracellular non-GPCR proteins affects various signaling pathways [80,85-89]. This includes inflammation [85,86], cell motility [81,90], and cell cycle [91,92] (Table 1). However, it remains unclear whether these atypical signaling pathways have physiological significance in vivo.

**Table 1:** Interactions of each GRKs with intracellular proteins

**GRK isoform**	**Binding partner**	**Function**	**Reference**
GRK2	Gα_q_	Regulation of Gα_q_ signaling	[27,28]
	mGluR1	Regulation of G protein signaling in a phosphorylation-independent manner	[29]
	Gβγ	Regulation of Gβγ-stimulated signaling	[31]
	PDGFRβ	Phosphorylation of PDGFRβ by GRK2 reduces PDGFRβ signaling	[78,79]
	HDAC6	GRK2 associates with and phosphorylates HDAC6 to enhance α-tubulin deacetylase activity and cell motility	[81]
	Akt	Interaction of GRK2 with Akt inhibits Akt activity	[82]
	p38	Phosphorylation of p38 by GRK2 impairs MKK6-induced p38 activation	[88]
	APC	Interaction of GRK2 with APC inhibits canonical Wnt signaling	[89]
	GIT1	Interaction between GRK2 and GIT1 is important for GRK2-mediated cell motility	[90]
	CDK2	Phosphorylation of GRK2 by CDK2 is important for cell cycle progression	[91]
	MEK	GRK2 negatively regulates CC chemokine ligand 2-induced ERK activation by the interaction with MEK	[93]
	IRS-1	Phosphorylation of IRS-1 by GRK2 mediates endothelin-1-induced insulin resistance	[94]
	clathrin	Interaction of GRK2 with clathrin promotes GPCR internalization	[95]
	PI3K	Translocation of PI3K to the plasma membrane is involved in GPCR internalization	[96]
	HSP90	Interaction of GRK2 with HSP90 at the mitochondria promotes pro-death signaling after ischemic injury	[100]
GRK5	β-arrestin1	Phosphorylation of β-arrestin1 by GRK5 down-regulates G protein-independent signaling	[80]
	HDAC5	Phosphorylation of HDAC5 promotes maladaptive cardiac hypertrophy	[83]
	p105	Interaction with p105 results in inhibition of lipopolysaccharide-induced ERK activation	[84]
	IκBα	Regulation of NF-κB signaling	[85,86]
	γ-tubulin centrin pericentrin	Co-localization of GRK5 with γ-tubulin, centrin, and pericentrin is important for regulation of microtubule nucleation and cell cycle progression	[92]
	p53	Phosphorylation of p53 by GRK5 inhibits DNA damage-induced apoptosis	[106]
	raptor	Grk5l, which is the closest homolog of GRK5 in zebrafish, interacts with raptor, and regulates mTOR signaling	[108]
GRK6	GIT1	GRK6 cooperates with GIT1 to enhance Rac1 activity, and promotes engulfment of apoptotic cells	[43]

Several reports have suggested that the interaction of GRK with intracellular non-GPCRs affects signaling pathways. It has been reported that GRK2 negatively regulates CCL2-induced ERK activation by interacting with mitogen-activated protein kinase kinase (MEK) [93]. Other signaling pathways, including the nuclear factor-kappa B (NF-κB) pathway [85,86], insulin signaling [94], and Smad signaling [87], have also been modulated by the interaction of GRKs with non-GPCR proteins. Although GRKs exhibit kinase activity, GRKs can interact with intracellular proteins and modulate downstream signaling pathways in a kinase activity-independent manner [95-97], indicating that GRKs can act as scaffold proteins. Because GRKs are composed of several domains other than a kinase domain, these regulatory domains may determine phosphorylation-independent signaling of GRKs.

Interactions between GRKs and intracellular proteins occurred at various sites including the outer membrane of the mitochondria and nucleus in addition to the plasma membrane and cytosol. For example, GRK2 was shown to localize in the mitochondria [98] and to interact with heat shock protein 90, a known mitochondrial chaperone [99]. A recent study further revealed that the ERK-mediated phosphorylation of GRK2 at Ser670 was important for the localization of GRK2 in the mitochondria, and this localization induced Ca^2+^-induced opening of the mitochondrial permeability transition pore after ischemic injury, which promoted cardiomyocyte death [100]. It was also shown that GRK2 was detected in the damaged mitochondria in the brain [101]. These reports suggested the crucial role of GRK2 in the mitochondria. In contrast, GRK5 was shown to localize in the nucleus and phosphorylated class II histone deacetylase 5 (HDAC5) [83]. This phosphorylation enhanced HDAC5 activity, leading to the export of HDAC from the nucleus. This resulted in the induction of myocyte enhancer factor-2 derepression and maladaptive cardiac hypertrophy. GRK5 was also reported to interact with the inhibitor of kappa B alpha (IκBα). Interaction between GRK5 and IκBα promoted the nuclear accumulation of IκBα, which resulted in the inhibition of NF-κB activity [86]. However, another group reported opposite results and showed that GRK5 enhanced NF-κB activity by promoting the phosphorylation and degradation of IκBα [85]. The NLS of GRK5 was important for nuclear function of GRK5. Therefore, other GRKs such as GRK4 and GRK6 (the GRK4 subfamily) may have similar functions in the nucleus as GRK5 because they also have their own NLS [40].

Some studies have reported the mechanism by which GRKs are activated and promote signaling by non-GPCR proteins [30]. It has been shown that GRK2 or GRK5 phosphorylates tubulin [102-104], and the phosphorylation level of tubulin by GRK2 is increased by β-AR stimulation [103]. Furthermore, GRK2 also phosphorylates insulin receptor substrate (IRS)-1, the phosphorylation activity of which is regulated by endothelin-1, an agonist of endothelin type A receptor [94]. These reports suggest that the binding of GRKs to activated GPCR could promote the interaction with intracellular non-GPCR proteins and stimulate the GRK-catalyzed phosphorylation of intracellular non-GPCR proteins. Participation of GRK2 in cellular regulation is also modulated by another kinase. The phosphorylation of GRK2 by cyclin-dependent kinase 2 (CDK2) transiently downregulates GRK2 expression, and the CDK2-catalyzed phosphorylation of GRK2 affects cell cycle progression [91]. In addition to phosphorylation, Cys of GRK2 at position 340 is modified by nitric oxide (NO), and the S-nitrosylation of GRK2 is critical for the downregulation of β-AR signaling in vitro and in vivo [105]. A cell-permeable NO donor, S-nitrosocysteine (CysNO), downregulated β-AR signaling by inhibiting the GRK2-catalyzed phosphorylation of β-AR and binding of β-arrestin to β-AR. Thus, posttranslational modification of GRKs may be another important factor for the regulation of GRK-mediated signaling.

Recent studies have suggested an in vivo significance of the interaction between GRK and intracellular non-GPCR proteins. GRK2 interacts with Akt and inhibits endothelial NO synthase activity and NO production, resulting in less severe portal hypertension in GRK2-deficient mice after liver injury [82]. GRK5 phosphorylates p53 and inhibits DNA damage-induced apoptosis in vitro and in vivo [106]. Although the mechanism is unknown, GRK2 was recently found to be involved in developmental and tumoral vascularization in mice [107]. That study was performed using endothelium-specific *Grk2*-knockout mice [107] because global ablation of GRK2 resulted in embryonic lethality [42]. Furthermore, it was recently revealed that Grk5l, which is the closest homolog of GRK5 in zebrafish, controlled heart formation during early development [108]. In their report, Grk5l was found to interact with Raptor, which is a component of mammalian target of rapamycin (mTOR) complex 1. Subsequently, the interaction of Grk5l with Raptor inhibited mTOR signaling by an unknown mechanism. Further studies are required to reveal undefined in vivo functions of GRKs with new binding partners.

Although the abovementioned studies have mainly focused on GRK2 and GRK5, the importance of the interaction of other GRK subfamilies with intracellular proteins remains poorly understood. GRK6 was recently found to mediate the removal of apoptotic cells (engulfment) and the clearance of senescent red blood cells through a new engulfment pathway [43]. Insufficient engulfment in GRK6-deficient mice resulted in the development of an autoimmune disease-like phenotype [43].

## Conclusions

It has become clear that GRKs are multifunctional proteins that interact not only with GPCRs but also with intracellular non-GPCR proteins. However, several issues remain to be resolved in future studies. One issue is the mechanism by which GRKs phosphorylate specific serine/threonine residues in GPCRs and non-GPCR proteins. Although GRKs can phosphorylate a large number of proteins, the consensus sequence of the phosphorylation site for each GRK has not been firmly established [109]. The second issue is the identification of molecules upstream of GRKs that are responsible for the increased phosphorylation of non-GPCR proteins. It is also important to elucidate signaling cascades from GRKs to cellular events. Another issue is the mechanisms for regulating the expression and activity of each GRK. We found that GRK6 expression was increased in MRL/*Lpr* mice, a murine model of systemic lupus erythematosus (SLE), and the autopsied spleens from SLE patients [43]. The changes in expression levels of GRKs were also found in patients with heart failure [110], schizophrenia [111], and depression [112]. However, it is unknown how these changes in expression cause these diseases. In contrast, it was revealed that overexpression of GRK2ct (also known as β-ARKct), a peptide inhibitor composed of the last 194 amino acids of GRK2, was successful for the prevention of heart failure through the inhibition of mitochondrial translocation [9,113-115]. These studies suggested that the inhibitors of GRKs could be effective for the treatment of heart failure [116]. Instead of the peptide inhibitor GRK2ct, chemical compounds are a more promising tool for treating heart failure. Recent reports revealed that the development of selective inhibitors against GRK2 is possible [117,118]. It is interesting to examine whether selective inhibition of GRK2 using chemical compounds [117,118] is beneficial for the abovementioned diseases.

## Competing interests

The authors declare that they have no competing interests.

## Authors’ contributions

KW wrote a draft, and MN and HK edited it. All authors read and approved the final manuscript.
